# Chemical synthesis using enzymatically generated building units for construction of the human milk pentasaccharides sialyllacto-*N*-tetraose and sialyllacto-*N*-neotetraose epimer

**DOI:** 10.3762/bjoc.6.18

**Published:** 2010-02-22

**Authors:** Dirk Schmidt, Joachim Thiem

**Affiliations:** 1 Faculty of Science, Department of Chemistry, University of Hamburg, Martin-Luther-King-Platz 6, D-20146 Hamburg, Germany

**Keywords:** block synthesis, human milk oligosaccharides, sialyllacto-*N*-neotetraose epimer, sialyllacto-*N*-tetraose, trisaccharide thioglycoside donors

## Abstract

α,2-3- and α,2-6-sialylated lactosaminide precursor structures obtained by various enzymatic procedures could be used for glycosylations employing triflic acid/*N*-iodosuccinimide. Easily accessible selectively protected lactoside derivatives served as acceptor disaccharides to give the corresponding human milk pentasaccharides in good yields. These were characterized by spectroscopic means in the form of their peracetylated derivatives.

## Introduction

From an inspection of contemporary syntheses of biologically and medicinally relevant oligosaccharides, it is evident that the majority is performed either by classical chemical methods or exclusively by enzymatic procedures. Even although considerable progress has been reported during the recent decades, every synthesis of a complex heterooligosaccharide still represents a challenge. To arrive at an oligosaccharide structure with specific patterns of substitution and defined regio- and stereochemical layout, all the presently available procedures need to be checked for efficiency with respect to not only all the above points, but also the efforts required at the purification steps as well as the yields. Whilst both approaches can be employed advantageously in certain cases, in others this is certainly not so.

For instance, for structures that contain glycosamino units, it has been demonstrated that these units can be introduced by classical methods in high yields and with good to excellent stereochemical control. In case of glucosylations and galactosylations, and in particular for β-galactosylations, classical and enzymatic methods are almost equal in terms of stereoselectivity and transfer efficiency. By contrast, for syntheses of sialylated structures, enzymatic procedures are still considerably superior to classical chemical sialylations with respect to both stereochemical outcome and preparative input.

The use of both procedures in a synergistic mode should also be considered. One of the general approaches ideally suited in such cases is the block synthesis method. Moreover, in recent years a number of combined chemical and chemoenzymatic syntheses have been reported [1–4].

**Figure 1:**
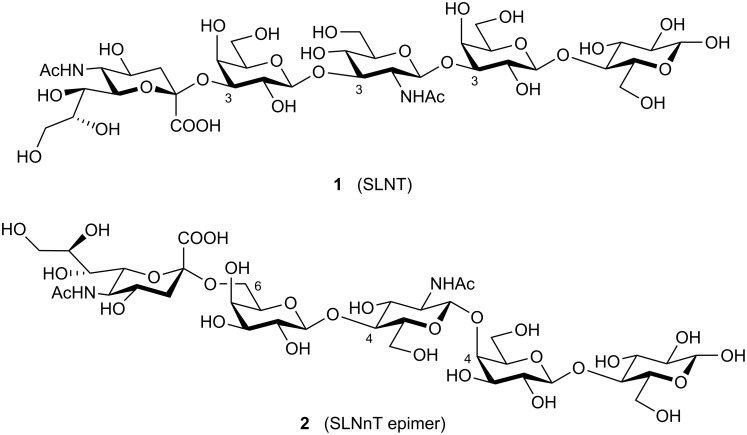
Structures of pentasaccharides **1** and **2**.

As a proof of principle, we were interested to employ some trisaccharide building units previously obtained by enzymatic routes in block syntheses *en route* to interesting structures. To this end, two human milk pentasaccharides of prominent importance, sialyllacto-*N*-tetraose (**1**) and an epimer of sialyllacto-*N*-neotetraose (**2**) (Figure 1) were selected as target molecules. Both these pentasaccharides, Neu5Acα2-3Galβ1-3GlcNAcβ1-3Galβ1-4Glc (**1**) and Neu5Acα2-6Galβ1-4GlcNAcβ1-4Galβ1-4Glc (**2**), are dominant constituents of complex human milk oligosaccharides (Figure 1). They are considered to play a major role in immuno defense against bacterial and viral infections in the gastrointestinal tract of infants [5]. It is thought that they effectively inhibit bacterial adhesion to epithelial surfaces and so block the first stages of infection processes. Thus, these human milk oligosaccharides are considered as soluble receptor analogues of epithelial cell surfaces [6].

## Results and Discussion

Previously, we reported the chemoenzymatic synthesis of the 3-sialylated lactosamine derivative **3** obtained by the enzymatic β-galactosylation of the 2-azidothioglucoside with *p*-nitrophenyl β-galactopyranoside and β-galactosidase (*Bovin testes*). The subsequent transsialylation was carried out with *p*-nitrophenyl sialoside (pNp-αNeu5Ac) and either sialidase from *Salmonella typhimurium* or from Newcastle disease virus [7]. Recently, a more effective higher yielding transfer has been reported in which sialylation with recombinant transsialidase (*Trypanosoma cruzi*) gave the trisaccharide **3** in 32% yield [8]. Treatment of **3** with methanol and acidic ion exchange resin led to the methyl ester (for the method cf. lit. [9]) which was then peracetylated to give trisaccharide **4** as the donor building block.

For formation of the disaccharide acceptor **6**, a straight-forward three-step standard reaction sequence was used [10]. Methyl β-lactoside was isopropylidenated at 3′,4′-position with dimethoxypropane and *p*-toluene sulfonic acid in DMF/acetone. Peracetylation (Ac_2_O/Py) and subsequent cleavage of the isopropylidene group with 80% acetic acid at 80 °C gave the diol acceptor **6**. Since it is known that in galactopyranosyl structures the nucleophilicity of 3-OH considerably exceeds that of the 4-OH-group, further protecting group manipulations were not required.

**Scheme 1:**
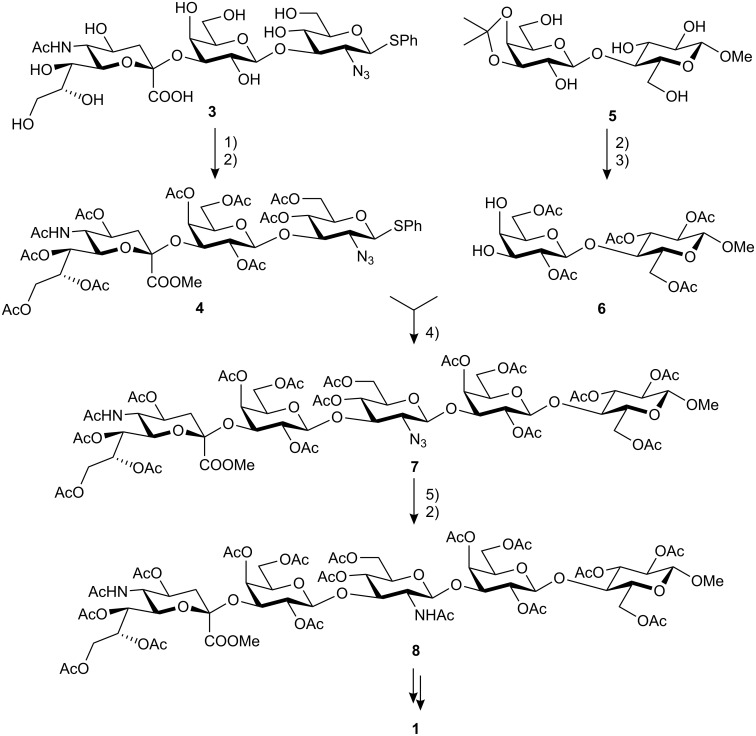
Preparation of pentasaccharide **8**. 1) MeOH, acidic ion exchange resin; 2) Ac_2_O, pyridine; 3) 80% HOAc, 90 °C; 4) NIS, CF_3_SO_3_H, 61%; 5) NiCl_2_•6H_2_O, H_3_BO_3_, EtOH, then NaBH_4_, EtOH and acidic workup.

Glycosylation of **4** by **6** catalyzed by *N*-iodosuccinimide and trifluoromethane sulfonic acid (as introduced by van Boom et al. [11]) gave the β,1-3-linked pentasaccharide **7** in 61% yield. About 5% of the corresponding α,1-3-linked compound and ca. 7% of the bis (β,1-3- and β,1-4-) linked octasaccharide were observed as side products and separated by chromatography but these were not further characterized. Reduction of the 2′′′-azido to the 2′′′-amino group with nickel boride [12–13] followed by peracetylation gave **8** in 81% yield (Scheme 1). ^1^H NMR spectrum contained a doublet for H-1″ at δ 4.96 (*J*_1″2″_ = 8.0 Hz) and a down field shifted doublet for H-4′ at δ 5.37 (*J*_3′4′_ = 2.9 Hz).

A similar approach was employed for the synthesis of the protected epimer of sialyllacto-*N*-neotetraose **14**. β-Galactosylation of 2-azidothioglucoside with *p*-nitrophenyl β-galactopyranoside and β-galactosidase (*Bacillus circulans*) gave the β,1-3-linked isolactosamine derivative. Further sialylation at position 3′-OH with pNp-αNeu5Ac and either sialidase from *Vibrio cholerae* or *Clostridium perfringens* afforded the α,2-6-sialylated trisaccharide **9** exclusively [7]. Later studies showed that **9** could be obtained in an enhanced yield of 32% by transsialylation with recombinant transsialidase (*Trypanosama cruzi*) [8]. Formation of the methyl ester and peracetylation led to the trisaccharide donor building block **10**.

Synthesis of the disaccharide acceptor in this case started from methyl β-lactoside, which was transformed into its 4′,6′-benzylidene-protected derivative **11** in almost quantitative yield by transacetalization with benzaldehyde dimethylacetal in acetonitrile under *p*-toluenesulfonic acid catalysis. Subsequent peracetylation with acetic anhydride/pyridine, selective cleavage of the benzylidene group with 80% acetic acid at 90 °C and finally treatment with *tert*-butyldiphenylsilyl chloride and imidazole in DMF afforded the disaccharide **12** (cf. references [14–15]).

After activation of the trisaccharide donor **10** with *N*-iodosuccinimide and trifluoromethanesulfonic acid, the disaccharide acceptor unit **12** could be glycosylated to give the β,1-4-linked pentasaccharide derivative **13** in 53% yield. In addition, the corresponding α,1-4-linked pentasaccharide was obtained in 8% yield.

**Scheme 2:**
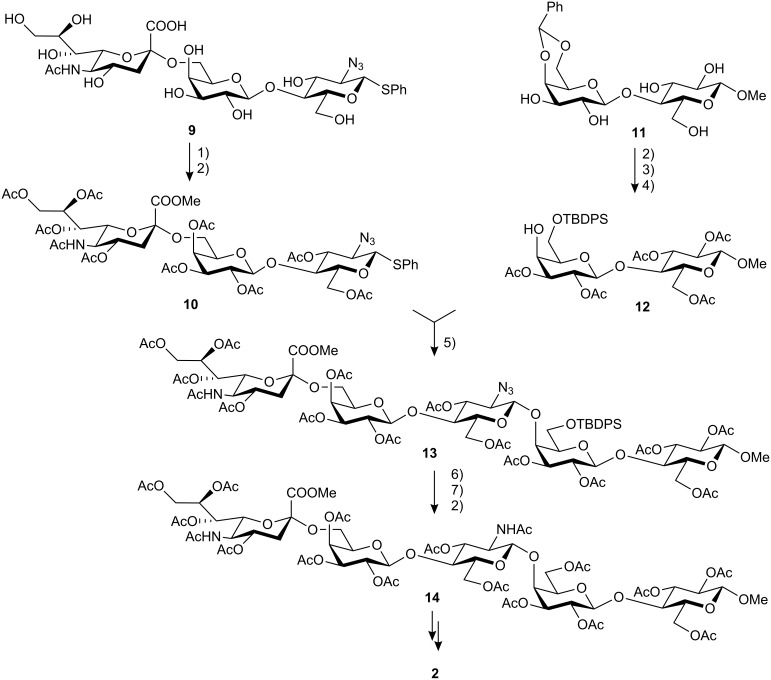
Preparation of pentasaccharide **14**. 1) MeOH, acidic ion exchange resin; 2) Ac_2_O, pyridine; 3) 80% HOAc, 90 °C; 4) TBDPSCl, imidazole, DMF; 5) NIS, CF_3_SO_3_H, 53%; 6) NiCl_2_•6H_2_O, H_3_BO_3_, EtOH, then NaBH_4_, EtOH, then acidic workup; 7) CF_3_CO_2_H, CH_2_Cl_2_.

Finally, the azido group was reduced by the nickel boride method with sodium borohydride, nickel chloride and boric acid [12–13]. During this step partial cleavage of the *tert*-butyldiphenylsilyl groups was also observed. Complete removal was achieved with trifluoroacetic acid in dichloromethane. For characterization purposes, peracetylation was carried out to give the completely protected pentasaccharide **14** in 67% yield (Scheme 2). As evident from a comparison of the ^1^H NMR data of **14** with the precursor tri- and disaccharide units **10** and **12**, the novel characteristic doublet for the anomeric H-1″ of the β-GlcNAc unit at δ 5.12 (*J*_1″2″_ = 8.2 Hz) as well as the downfield shift Δδ 0.15 of H-4′ to δ 4.14 compared to **12** were in accord with structure of the target pentasaccharide.

## Conclusion

In this contribution chemoenzymatically generated sialyl α,2-3- and sialyl α,2-6-glycosylated thiophenol 2-azido-lactose derivatives were employed as precursors for sialylated lactosaminide donor substituents in triflic acid/*N*-iodosuccinimide glycosylations. With easily accessible selectively unprotected lactose acceptor glycosides the pentasaccharide structures sialyllacto-*N*-tetraose and the epimer of sialyllacto-*N*-neotetraose could be obtained in good yields, and subsequently transformed into their peracetylated derivatives for structure elucidation. Thus, a combination of enzymatic and purely chemical procedures was shown to be advantageous in the preparation of complex oligosaccharides.

## Experimental

**Figure 2:**
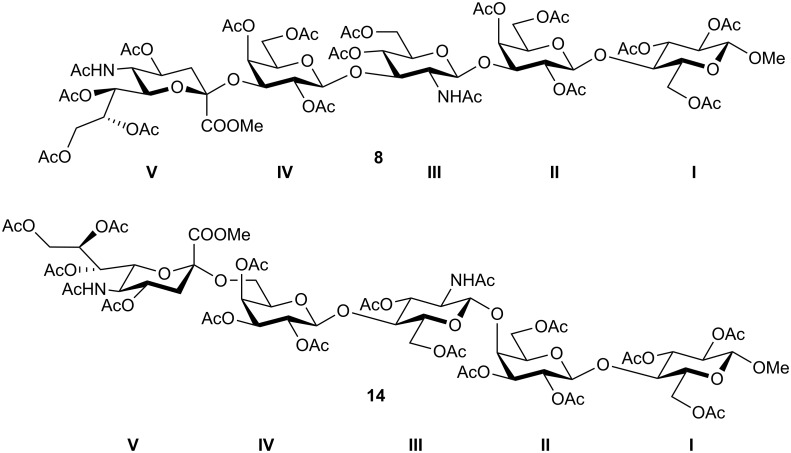
Roman numbering of saccharide units in all pentasaccharides for NMR assignment.

For general methods cf. reference [16]. The NMR data for the saccharide rings in the pentasaccharides **7**, **8**, **13** and **14** are denoted according to the Roman numberals I-V from the reducing end, as depicted for compounds **8** and **14** (Figure 2):

**Methyl *O*-(methyl-5-acetamido-4,7,8,9-tetra-*O*-acetyl-3,5-dideoxy-α-D-glycero-D-galacto-2-nonulopyranosylonate)-(2-3)-*O*-(2,4,6-tri-*O*-acetyl-β-D-galactopyranosyl)-(1-3)-*O*-(4,6-di-*O*-acetyl-2-azido-2-deoxy-β-D-glucopyranosyl)-(1-3)-*O*-(2,4,6-tri-*O*-acetyl-β-D-galactopyranosyl)-(1-4)-2,3,6-tri-O-acetyl-β-D-glucopyranoside (7):** Glycosylation was carried out as described for the synthesis of compound **13** from compound **4** (95 mg, 83 μmol) as donor and compound **6** (50 mg, 86 μmol) as acceptor. The pentasccharide derivative **7** was obtained as a colorless amorphous solid; 83 mg (61%); [α]_D_^20^ = −3.5 (*c* 0.1, CHCl_3_); ^1^H NMR (500 MHz, CDCl3) δ 5.75 (dt, 1 H, *H-*8^V^), 5.72 (d, 1 H, NH), 5.39 (d, *J*_3,4_ = 2.8 Hz, 1 H, *H*-4^II^), 5.36 (dd, *J*_7,8_ = 9.3 Hz, 1H, *H*-7^V^), 5.15 (dd, J_2,3_ = *J*_3,4_ = 9.4 Hz, 1 H, *H*-3^I^), 5.06 (dd, *J*_1,2_ = 8.0 Hz, *J*_2,3_ = 10.2 Hz, *H*-2^IV^), 4.99–4.97 (m, 2 H, *H*-2^II^, *H*-4^III^), 4.96 (dd, *J*_1,2_ = 7.8 Hz, *J*_2,3_ = 9.6 Hz, 1 H, *H*-2^I^), 4.91 (dd, *J*_1,2_ = 8.0 Hz, *J*_2,3_ = 10.2 Hz, 1 H, *H*-1^IV^), 4.87 (d, *J*_3,4_ = 2.6 Hz, *H*-4^IV^), 4.83 (dt, *J*_3eq,4_ = 4.4 Hz, *J*_3ax,4_ = *J*_4,5_ = 12.1 Hz, 1 H, *H*-4^V^), 4.75 (dd, *J*_5,6_ = 10.1 Hz, J_6,7_ = 2.2 Hz, *H*-6^V^), 4.62 (dd, 1 H, *H*-3^III^), 4.57 (dd, *J*_2,3_ = 10.0 Hz, *J*_3,4_ = 2.8 Hz, 1 H, *H*-3^IV^), 4.51 (d, *J*_1,2_ = 7.8 Hz, 1 H, *H*-1^I^), 4.49 (dd, 1 H, *H*-6^V^), 4.39 (d, *J*_1,2_ = 8.0 Hz, 1 H, *H*-1^II^), 4.37 (d, *J*_1,2_ = 8.1 Hz, 1 H, *H*-1^III^), 4.29 (dd, *J*_9a,9b_ = 12.4 Hz, *J*_8,9b_ = 2.4 Hz, *H*-9b^V^), 4.24 (dd, *J*_6a,6b_ = 12.6 Hz, *J*_5,6a_ = 6.6 Hz, 1 H, *H*-6a^III^), 4.17 (dd, *J*_5,6_ = 10.2 Hz, *H*-5^V^), 4.13 (d, 1 H, NH), 3.75 (s, 3 H, COOCH_3_^V^), 4.00 (dd, *J*_8,9a_ = 5.8 Hz, 1 H, *H*-9a^V^), 3.72 (bt, 1 H, *H*-5^IV^), 3.59 (m, 1 H, *H*-5^I^), 3.47–3.44 (m, 2 H, *H*-3^II^, *H*-6a^IV^), 3.39 (dd, *J*_6a,6b_ = 11.0 Hz, *J*_5,6b_ = 7.8 Hz, *H*-6b^IV^), 2.61 (dd, *J*_3ax,3eq_ = 12.4 Hz, *J*_3eq,4_ = 4.8 Hz, 1 H, *H*-3eq^V^), 2.17–1.33 (15s, 45 H, 14 OAc, 1 NAc), 1.91 (dd, J_3ax,4_ = 11.8 Hz, 1 H, *H*-3ax^V^). C_67_H_92_N_4_O_43_ (1641.45): Found C, 49.33; H, 5.59; N, 3.62. Calculated C; 49.02; H, 5.65; N, 3.41. MALDI-TOF: 1664.44 (M+Na)^+^; 1680.59 (M+K)^+^.

**Methyl *O*-(methyl-5-acetamido-4,7,8,9-tetra-*O*-acetyl-3,5-dideoxy-α-D-glycero-D-galacto-2-nonulopyranosylonate)-(2-3)-*O*-(2,4,6-tri-*O*-acetyl-β-D-galactopyranosyl)-(1-3)-*O*-(4,6-di-*O*-acetyl-2-acetamido-2-deoxy-β-D-glucopyranosyl)-(1-3)-*O*-(2,4,6-tri-*O*-acetyl-β-D-galactopyranosyl)-(1-4)-2,3,6-tri-O-acetyl-β-D-glucopyranoside (8):** Reduction and peracetylation of compound **7** was carried out as described for **14**. Thus, from 80 mg (49 μmol) of **7**, 65 mg (81%) of **8** was obtained as a colorless amorphous solid; [α]_D_^20^ = −12.7 (*c* 0.5, CHCl_3_); ^1^H NMR (500 MHz, CDCl_3_) δ 5.74 (dt, 1 H, *H-*8^V^), 5.72 (d, 1 H, NH), 5.38 (d, *J*_3,4_ = 2.8 Hz, 1 H, *H-*4^II^), 5.33 (dd, *J*_7,8_ = 9.3 Hz, 1H, *H-*7^V^), 5.12 (dd, *J*_2,3_ = *J*_3,4_ = 9.6 Hz, 1 H, *H*-3^I^), 5.05 (dd, *J*_1,2_ = 8.0 Hz, *J*_2,3_ = 10.2 Hz, *H*-2^IV^), 4.99 (dd, *J*_1,2_ = 8.0 Hz, J_2,3_ = 9.7 Hz, 1 H, *H*-2^II^), 4.97 (m, 2 H, *H*-4^III^), 4.95 (dd, *J*_1,2_ = 8.0 Hz, *J*_2,3_ = 9.8 Hz, 1 H, *H*-2^I^), 4.91 (dd, *J*_1,2_ = 7.9 Hz,1 H, *H*-1^IV^), 4.86 (dt, *J*_3eq,4_ = 4.6 Hz, *J*_3ax,4_ = *J*_4,5_ = 12.0 Hz, 1 H, *H*-4^V^), 4.83 (d, *J*_3,4_ = 2.6 Hz, *H*-4^IV^), 4.75 (dd, *J*_5,6_ = 9.9 Hz, *J*_6,7_ = 1.8 Hz, *H*-6^V^), 4.61 (dd, 1 H, *H-*3^III^), 4.57 (dd, *J*_2,3_ = 9.8 Hz, *J*_3,4_ = 2.6 Hz, 1 H, *H*-3^IV^), 4.53 (dd, 1 H, *H*-6^I^), 4.51 (d, *J*_1,2_ = 8.0 Hz, 1 H, *H*-1^I^), 4.39–4.37 (m, 2 H, *H*-1^II^, *H*-1^III^), 4.32 (dd, *J*_9a,9b_ = 12.3 Hz, *J*_8,9b_ = 2.1 Hz, *H*-9b^V^), 4.26 (dd, *J*_6a,6b_ = 12.2 Hz, *J*_5,6a_ = 6.1 Hz, 1 H, *H-*6a^III^), 4.13 (dd, *J*_5,6_ = 9.8 Hz, *H*-5^V^), 4.13 (d, 1 H, NH), 4.00 (dd, *J*_8,9a_ = 5.8 Hz, 1 H, *H*-9a^V^), 3.75 (s, 3 H, COOCH_3_^V^), 3.71 (bt, 1 H, *H*-5^IV^), 3.55 (m, 1 H, *H*-5^I^), 3.46–3.44 (m, 2 H, *H*-6a^IV^, *H*-3^II^), 3.43 (dd, *J*_6a,6b_ = 11.0 Hz, *J*_5,6b_ = 7.8 Hz, *H*-6b^IV^), 2.59 (dd, *J*_3ax,3eq_ = 12.2 Hz, *J*_3eq,4_ = 4.6 Hz, 1 H, *H*-3eq^V^), 2.21–1.35 (17s, 51 H, 15 OAc, 2 NAc), 1.93 (dd, *J*_3ax,4_ = 12.0 Hz, 1 H, *H*-3ax^V^). C_69_H_96_N_2_O_44_ (1657.49): Found C, 49.90; H, 5.69; N, 1.57. Calculated C, 50.00; H, 5.84; N, 1.69. MALDI-TOF: 1680.48 (M+Na)^+^; 1696.59 (M+K)^+^.

**Methyl *O*-(methyl-5-acetamido-4,7,8,9-tetra-*O*-acetyl-3,5-dideoxy-α-D-glycero-D-galacto-2-nonulopyranosylonate)-(2-6)-*O*-(2,3,4-tri-*O*-acetyl-β-D-galactopyranosyl)-(1-4)-*O*-(3,6-di-*O*-acetyl-2-azido-2-deoxy-β-D-glucopyranosyl)-(1-4)-*O*-(2,3-di-*O*-acetyl-6-*O-tert*-butyldiphenylsilyl-β-D-galactopyranosyl)-(1-4)-*O*-2,3,6-tri-*O*-acetyl-β-D-glucopyranoside (13):** A solution of trisaccharide **10** (68 mg, 60 μmol) and disaccharide **12** (56 mg, 70 μmol) in anhydrous toluene (2 mL) was cooled to −40 °C. *N*-Iodosuccinimide (20 mg, 94 μmol), molecular sieves (4 Å, 200 mg) were added, and after cooling a saturated solution of trifluoromethane sulfonic acid in CCl_4_ (ca. 2 M, 50 μL) was added with vigorous stirring. The mixture was gradually warmed over 2.5 h to −10 °C. Ethyl acetate (20 mL) was added and the reaction quenched by addition of a saturated aqueous NaHCO_3_ solution (10 mL). After filtration through Celite, the phases were separated. The organic phase was washed with aqueous Na_2_S_2_O_3_ solution (10 mL), dried over MgSO_4_, evaporated and the residue purified by flash chromatography with petroleum ether/ethyl acetate 2:1. Compound **7** was obtained as a colorless amorphous solid; 58 mg (53%). [α]_D_^20^ = −21.6 (*c* 0.3, CHCl_3_); ^1^H NMR (500 MHz, CDCl_3_) δ 7.70–7.24 (m, 10, Ph), 5.74 (dd, 1 H, *H*-4^IV^), 5.70 (ddd, 1 H, *H*-8^V^), 5.61 (dd, *J*_2,3_ = 10.2 Hz, 1 H, *H*-2^IV^), 5.44 (dd, *J*_7,8_ = 9.0 Hz, 1 H, *H*-7^V^), 5.26 (dd, 1 H, *H*-3^IV^), 5.22 (t, *J*_2,3_ = *J*_3,4_ = 9.8 Hz, 1 H, *H*-3^I^), 5.10 (dd, *J*_1,2_ = 8.0 Hz, *J*_2,3_ = 9.9 Hz, 1 H, *H*-2^II^),4.93 (t, *J*_2,3_ = 9.8 Hz, 1 H, *H*-2^I^), 4.87 (2d, 2 H, *H*-1^IV^, *H*-3^III^), 4.85 (ddd, *J*_4,5_ = 10.2 Hz, 1 H, *H*-4^V^), 4.65 (dd, 1 H, *H*-3^II^), 4.61 (d, *J*_3,4_ = 4.0 Hz, *H*-4^II^), 4.59 (dd, *J*_9a,9b_ = 12.2 Hz, 1 H, *H*-9a^V^), 4.51 (d, 1 H, *H*-1^III^), 4.49 (d, *J*_1,2_ = 8.0 Hz, 1 H, *H*-1^II^), 4.48 (dd, 1 H, *H*-6b^III^), 4.46 (d, *J*_1,2_ = 10.0 Hz, 1 H, *H*-1^I^), 4.42 (dd, 1 H, *H*-6b^I^), 4.34 (dd, 1 H, *H*-9b^V^), 4.32 (d, 1 H, N*H*^V^), 4.29 (ddd, *J*_5,6_ = 10.6 Hz, 1 H, *H*-5^V^), 4.20 (dd, 1 H, *H*-6b^III^), 4.13 (m, 2 H, *H*-6^V^, *H*-6b^II^), 4.10 (dd, *J*_5,6a_ = 5.6 Hz, *J*_6a,6b_ = 12.4 Hz, 1 H, *H*-6a^I^), 4.08 (dd, *J*_5,6a_ = *J*_6a,6b_ = 6.0 Hz, 1 H, *H*-6a^II^), 4.06 (dd, 1 H, *H*-6b^IV^), 3.88 (ddd, 1 H, *H*-5^IV^), 3.83 (m, 2 H, *H*-5I, *H*-4^III^), 3.78 (s, 3 H, OCH_3_), 3.77 (t, *J*_3,4_ = 9.9 Hz, 1 H, *H*-4^I^), 3.62 (ddd, *J*_4,5_ = 10.0 Hz, *J*_5,6a_ = 5.5 Hz, *J*_5,6b_ = 2.0 Hz, 1 H, *H*-5^I^), 3.61 (dd, 1 H, *H*-6b^IV^), 3.59 (ddd, 1 H, *H*-5^III^), 3.56 (dd, *J*_2,3_ = 10.2 Hz, 1 H, *H*-2^III^), 3.33 (s, 3 H, C*H*_3_^V^), 2.69 (dd, *J*_3eq_,3_ax_ = 12.7 Hz, *J*_3eq,4_ = 4.6 Hz, 1 H, *H*-3_eq_^V^), 2.15–1.36 (14s, 42 H, 13 OAc, 1 NAc), 2.03 (dd, *J*_3ax,4_ = 12.0 Hz, 1 H, *H*-3_ax_^V^), 1.01 (s, 9 H, SiCCH_3_). C_81_H_108_N_4_O_42_Si (1837.81): Found C, 53.89; H, 6.34; N, 2.66. Calculated C, 52.94; H, 5.92; 3.04. MALDI-TOF: 1860.80 (M+Na)^+^; 1876.91 (M+K)^+^. The α,1^III^-4^II^-anomer of **7** was obtained as colorless syrup (9 mg, 8%) and not further characterized.

**Methyl *O*-(methyl-5-acetamido-4,7,8,9-tetra-*O*-acetyl-3,5-dideoxy-α-D-*glycero*-D-*galacto*-2-nonulopyranosylonate)-(2-6)-*O*-(2,3,4-tri-*O*-acetyl-β-D-galactopyranosyl)-(1-4)-*O*-(3,6-di-*O*-acetyl-2-acetamido-2-deoxy-β-D-glucopyranosyl)-(1-4)-*O*-(2,3,6-tri-*O-*acetyl-β-D-galactopyranosyl)-(1-4)-*O*-2,3,6-tri-O-acetyl-β-D-glucopyranoside (14):** Compound **13** (53 mg, 29 μmol), NiCl_2_·6H_2_O (105 mg, 450 μmol) and boric acid (55 mg, 900 μmol) were dissolved in ethanol (3 mL). Under vigorous stirring a suspension of sodium borohydride (28 mg, 750 μmol) in ethanol (1 mL) was added with the temperature maintained at 20 °C. After 30 min ethanol (6 mL) and acetic acid (3 mL) were added. Then the mixture was co-distilled three times with toluene (5 mL each), and then the residue dissolved in dichloromethane (10 mL). After washing with diluted aqueous KHSO_4_ solution (5 mL), saturated aqueous NaHCO_3_ solution (5 mL), and water (5 mL), the organic phase was dried (MgSO_4_) and evaporated to dryness. The resulting material was treated with dichloromethane/trifluoroacetic acid (9:1, 2 mL) for 1 h at room temperature, then co-distilled three times with toluene (5 mL each) and dried under high vacuum. The residue was treated with acetic anhydride (1 mL) and pyridine (5 mL) for 10 h, then co-distilled three times with toluene (5 mL each). Purification by flash chromatography (toluene/acetone 3:1) gave **14** (35 mg, 67%) as a colorless amorphous solid; [α]_D_^20^ = −31.2 (*c* 0.4, CHCl_3_); ^1^H NMR (500 MHz, CDCl_3_) δ 7.72–7.25 (m, 10, Ph), 5.72 (ddd, 1 H, *H*-8^V^), 5.70 (dd, 1 H, *H*-4^IV^), 5.59 (dd, *J*_2,3_ = 9.9 Hz, 1 H, *H*-2^IV^), 5.43 (dd, *J*_7,8_ = 8.8 Hz, 1 H, *H*-7^V^), 5.32 (d, J_3,4_ = 4.0 Hz, *H*-4^II^), 5.23 (dd, 1 H, *H*-3^IV^), 5.21 (t, *J*_2,3_ = *J*_3,4_ = 10.2 Hz, 1 H, *H*-3^I^), 5.11 (dd, *J*_1,2_ = 7.8 Hz, *J*_2,3_ = 9.8 Hz, 1 H, *H*-2^II^), 4.90 (t, *J*_2,3_ = 10.0 Hz, 1 H, *H*-2^I^), 4.88 (d, 1 H, *H*-1^IV^), 4.86 (m, 2 H, *H*-3^III^, *H*-4^V^), 4.63 (dd, 1 H, *H*-3^II^), 4.59 (dd, *J*_9a,9b_ = 11.9 Hz, 1 H, *H*-9a^V^), 4.53 (d, *J*_1,2_ = 8.0 Hz, 1 H, *H*-1^II^), 4.50 (d, 1 H, *H*-1^III^), 4.47 (m, 2 H, *H*-6b^III^, *H*-1^I^), 4.39 (dd, 1 H, *H*-6b^I^), 4.32 (d, 1 H, N*H*), 4.31 (dd, 1 H, *H*-9b^V^), 4.27 (ddd, *J*_5,6_ = 10.3 Hz, 1 H, *H*-5^V^), 4.18 (dd, 1 H, *H*-6b^III^), 4.11 (dd, 1 H, *H*-6^V^), 4.09 (dd, *J*_5,6a_ = 5.8 Hz, *J*_6a,6b_ = 12.2 Hz, 1 H, *H*-6a^I^), 4.01 (dd, 1 H, *H*-6b^IV^), 3.92 (ddd, 1 H, *H*-5^IV^), 3.87 (dd, *J*_5,6a_ = J_6a,6b_ = 6.0 Hz, 1 H, *H*-6a^II^), 3.85 (dd, 1 H, *H*-6b^II^), 3.82 (m, 2 H, *H*-5^II^, *H*-4^III^), 3.77 (s, 3 H, OCH_3_), 3.75 (t, *J*_3,4_ = 10.1 Hz, 1 H, *H*-4^I^), 3.65 (ddd, *J*_4,5_ = 9.8 Hz, *J*_5,6a_ = 5.6 Hz, *J*_5,6b_ = 1.8 Hz, 1 H, *H*-5^I^), 3.59 (m, 2 H, *H*-6b^IV^, *H*-5^III^), 3.57(dd, *J*_2,3_ = 10.0 Hz, 1 H, *H*-2^III^), 3.31 (s, 3 H, C*H*_3_^V^), 2.70 (dd, *J*_3eq,3ax_ = 12.5 Hz, *J*_3e,4_ = 4.4, 1 H, *H*-3eq^V^), 2.17–1.33 (16s, 48 H, 14 OAc, 2 NAc), 2.01 (dd, *J*_3ax,4_ = 12.0 Hz, 1 H, *H*-3ax^V^), 1.00 (s, 9 H, SiCCH_3_). C_69_H_96_N_2_O_44_ (1657.49): Found C, 49.86; H, 5.77; N, 1.65. Calculated C, 50.00; H, 5.84; N, 1.69. MALDI-TOF: 1680.39 (M+Na)^+^; 1696.59(M+K)^+^.

## References

[1] Look G C, Ichikawa Y, Shen G-J, Cheng P-W, Wong C-H (1993). J Org Chem.

[2] Palcic M M, Li H, Zanini D, Bhella R S, Roy R (1998). Carbohydr Res.

[3] Unverzagt C (1994). Angew Chem, Int Ed Engl.

[4] Unverzagt C (1998). Carbohydr Res.

[5] Estabrook M M, Griffiss J M, Jarvis G A (1997). Infect Immun.

[6] Kunz C, Rudloff S (1996). Z Ernährungswiss.

[7] Schmidt D, Sauerbrei B, Thiem J (2000). J Org Chem.

[8] Neubacher B, Schmidt D, Ziegelmüller P, Thiem J (2005). Org Biomol Chem.

[9] Kuhn R, Lutz P, MacDonald D L (1966). Chem Ber.

[10] Zhu X X, Ding P, Cai M S (1996). Carbohydr Res.

[11] Veeneman G H, van Leeuwen S H, van Boom J H (1990). Tetrahedron Lett.

[12] Paulsen H, Sinnwell V (1978). Chem Ber.

[13] Thiem J, Sievers A (1979). Chem Ber.

[14] Zhang Z, Magnusson G (1995). J Org Chem.

[15] DeNinno M P, Eller C, Etienne J B (1998). Bioorg Med Chem Lett.

[16] Neumann J, Thiem J (2010). Eur J Org Chem.

